# *Scaldiporia vandokkumi*, a new pontoporiid (Mammalia, Cetacea, Odontoceti) from the Late Miocene to earliest Pliocene of the Westerschelde estuary (The Netherlands)

**DOI:** 10.7717/peerj.3991

**Published:** 2017-11-01

**Authors:** Klaas Post, Stephen Louwye, Olivier Lambert

**Affiliations:** 1 Natuurhistorisch Museum Rotterdam, Rotterdam, The Netherlands; 2 Paleontology Research Unit, Ghent University, Ghent, Belgium; 3 D.O. Terre et Histoire de la Vie, Institut royal des Sciences naturelles de Belgique, Brussels, Belgium

**Keywords:** Cetacea, Inioidea, Pontoporiidae, Westerschelde, The Netherlands, Late Miocene, earliest Pliocene

## Abstract

**Background:**

The family Pontoporiidae (Cetacea, Odontoceti, Inioidea) is currently represented in our oceans by just one species of diminutive dolphin (*Pontoporia blainvillei,* franciscana). Although *P. blainvillei* is limited to coastal waters of the South Atlantic along Brazil, Uruguay and Argentina, multiple Miocene and Pliocene fossils indicate the past presence of members of the family in the South Atlantic, South Paciifc and North Atlantic oceans. Our comprehension of the origin and diversity of this clade and of the relationships of its members with other inioids is hampered by the fact that part of the described fossil specimens, especially from the North Atlantic realm, are cranial fragments often associated to limited stratigraphic information.

**Methods:**

Based on an almost complete fossil cranium of pontoporiid from the Westerschelde estuary, The Netherlands, whose preservation allows for detailed morphological observations, we describe a new genus and species. The latter is compared to other pontoporiids, as well as a few non-pontoporiid inioids. A phylogenetic analysis is performed to investigate the relationship of *S. vandokkumi* with the best-known extinct and extant inioids. Palynological analysis of the sediment associated to the holotype is used to assess its geological age.

**Results and discussion:**

The new genus and species *Scaldiporia vandokkumi* is characterized among others by greatly thickened premaxillary eminences reaching the level of the antorbital notch. Palynologically dated from the late Tortonian—earliest Zanclean (7.6–5 Ma, Late Miocene—earliest Pliocene), this new pontoporiid confirms the surprising past diversity of marine inioids in the North Atlantic area. Finally the content of the pontoporiid subfamily Brachydelphininae is briefly discussed.

## Introduction

Publications of the last decades prove the living franciscana *Pontoporia blainvillei* (Gervais & D’Orbigny, 1848) and Amazon river dolphin *Inia geoffrensis* (Blainville, 1817) to be remnants of a species-rich Inioidea clade (*sensu* Muizon, 1988a), which left fossil traces from the South- and North Pacific to the North Atlantic (for a summary see: Cozzuol, 2010; Pyenson et al., 2015; Murakami, 2016; Lambert et al., 2017). While some extinct and extant freshwater species are recorded among Iniidae (Cozzuol, 2010; Gutstein, Cozzuol & Pyenson, 2014), all inioids attributed to the family Pontoporiidae are reported from marine environments. To date the following pontoporiid genera are recognised: the extinct *Auroracetus*, *Brachydelphis*, *Pliopontos*, *Pontistes*, *Protophocaena*, and *Stenasodelphis*, and the extant *Pontoporia*, (Burmeister, 1885; Muizon, 1984; Muizon, 1988b; Lambert & Post, 2005; Godfrey & Barnes, 2008; Gibson & Geisler, 2009; Lambert & Muizon, 2013). The anatomy of *Pontoporia blainvillei*, from the coastal waters of eastern South America is known in detail (Flower, 1867; Brownell Jr, 1989), and cranial and some postcranial remains of *Brachydelphis* and *Pliopontos*, from the southeast Pacific, are also well described. The North Atlantic, South Atlantic, and North Sea fossil pontoporiid taxa however are based on isolated single—or at most a few—cranial fragment(s), obstructing detailed descriptions and a thorough phylogenetic analysis of the family (Geisler, Godfrey & Lambert, 2012; Gutstein et al., 2009; Lambert & Muizon, 2013).

The tentative incorporation into one subfamily (Brachydelphininae Muizon, 1988a) of the South American *Brachydelphis* and the poorly known *Protophocaena* of the Late Miocene North Sea still needs to be corroborated (Geisler, Godfrey & Lambert, 2012; Lambert & Muizon, 2013; Lambert et al., 2017). The latter genus was initially described as a phocoenid based on a single fragmentary cranium (Abel, 1905), but was later recognised as a pontoporiid (Lambert & Post, 2005). These authors also suggested the probable existence in the North Sea of additional pontoporiid taxa based on morphometrics of fossil periotics from Belgium and The Netherlands. Later, unnamed incomplete pontoporiid crania from the Tortonian of Denmark (Pyenson & Hoch, 2007) and inioid crania from the southern margin of the North Sea (Post & Bosselaers, 2017) proved that an array of inioid taxa frequented the North Sea during the late Neogene.

The present article describes a new Late Miocene—earliest Pliocene pontoporiid from the southern margin of the North Sea. The fairly well preserved cranium—the most complete pontoporid specimen outside Peru and the most complete inioid specimen outside Latin America—allows a detailed description. This new taxon corroborates the past abundance of inioid taxa in the North Atlantic realm and underlines a further degree of morphological disparity within the superfamily.

## Material and Methods

**Nomenclatural acts**: The electronic version of this article in portable document format (PDF) will represent a published work according to the International Commission on Zoological Nomenclature (ICZN), and hence the new names contained in the electronic version are effectively published under that code from the electronic edition alone. This published work and the nomenclatural acts it contains have been registered in ZooBank, the online registration system for the ICZN. The ZooBank LSIDs (life science identifiers) can be resolved and the associated information viewed through any standard web browser by appending the LSID to the prefix http://zoobank.org/. The LSID for this publication is: urn:lsid:zoobank.org:pub:B5D0467C-E361-4278-9A8C-937B0DD11C39. The online version of this work is archived and available from the following digital repositories: PeerJ, PubMed Central and CLOCKSS.

**Figure 1:**
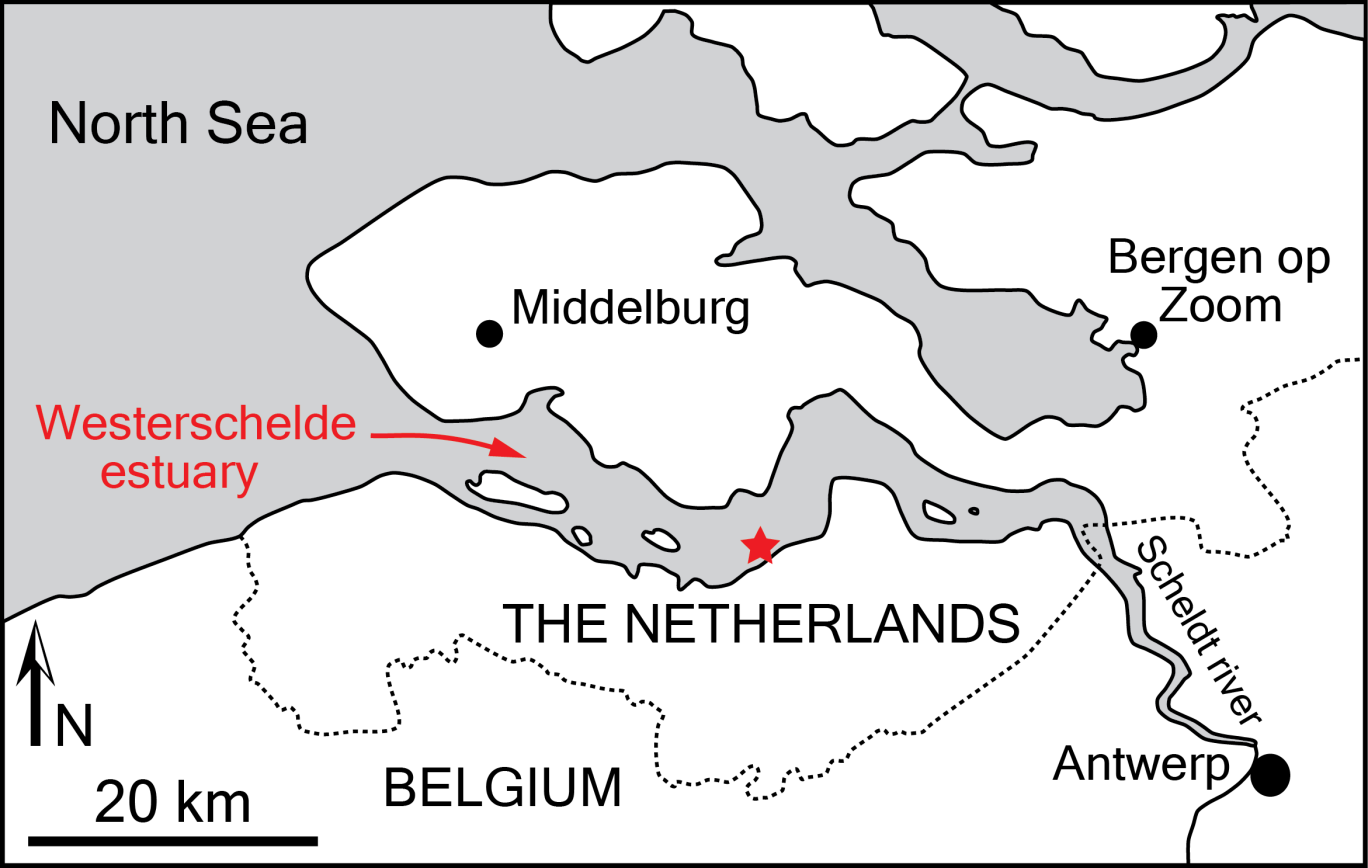
Locality of the holotype of *Scaldiporia vandokkumi*. Simplified map of the Belgium-Netherlands border area, showing the Westerschelde estuary with the position of the type locality of *S. vandokkumi* (red star), based on data from Post & Reumer (2016).

**Studied specimen**: The cranium NMR 9991-12018 was discovered by NMR expedition 2014-3 trawling the bottom of a specific small site in the Westerschelde estuary, The Netherlands (Post & Reumer, 2016; Fig. 1). The cranium was found embedded in a hard glauconitic sand matrix, which was removed by mechanical preparation.

**Other specimens directly observed**: *Brachydelphis mazeasi*: MNHN PPI 121, 124, 266, MUSM 564, 589, 591, 593, 886, 887, 2473, 2539; *B. jahuayensis*: MNHN PPI 267, 268, MUSM 567, 568, 712, 884, 2611 (=AGL 141), MUSM 2618 (=AGL 732); *Brujadelphis ankylorostris*: MUSM 1400; *Inia geoffrensis*: USNM 395614, ZMA 17.771, several unnumbered specimens at MNHN and MUSM; *Lipotes vexillifer*: USNM 218293; *Meherrinia isoni*: CMM-V-4051, 4052, 4060, IRSNB M.2013 *Parapontoporia sternbergi*: NMR 9992-3761; *Pliopontos littoralis*: MNHN SAS 193, 931, 953, MUSM 953, 976, 6253; cf. *Pontistes* sp.: cast of MGUH 1922-168; *Pontoporia blainvillei*: IRSNB 1506, NBC/ZMA 16.714 + one unnumbered specimen, MSM TP 273, USNM 482772; Pontoporiidae, gen. et sp. indet.: cast of MGUH 1910-274; *Protophocaena minima*: IRSNB 3917-M.172, M.2303, NMB 1, OMB 4704, TM 25112; *Stenasodelphis russellae*: CMM-V-2234.

**Anatomical terminology**: The terminology for cranial anatomy follows Mead & Fordyce (2009); exceptions are noted directly in the text.

**Phylogenetic analysis**: To analyse the phylogenetic relationships of the new taxon among delphinidans, we coded NMR 9991-12018 in the matrix of 324 morphological characters and 105 operational taxonomic units from Lambert et al. (2017) (see Supplemental Information). Using PAUP 4.0a (Swofford, 2003), three outgroups were a priori defined (*Bos taurus*, *Hippopotamus amphibius*, and *Sus scrofa*), ordered multistate characters were scaled for a minimum length of each being one step, and a constraint tree resulting from Bayesian analysis of molecular data on extant taxa was enforced as a backbone (see Supplemental Information), in the same way as in Lambert et al. (2017). Most parsimonious trees were obtained via heuristic search, using the tree-bisection-reconnection branch swapping algorithm and ACCTRAN character-state optimization.

**Palynological analysis**: A sediment sample of glauconitic sand was recovered from the cerebral cavity of the cranium NMR 9991-12018 and palynologically analysed for organic-walled dinoflagellate cysts (dinocysts) and acritarchs. The palynological preparation of the sediments followed standard techniques described by Louwye, Head & De Schepper (2004). The microscopic analysis was carried out with a transmitted light microscope Zeiss AxioImager A1 under a 400× magnification. The entire slide was scanned in non-overlapping traverses. The taxonomy of the dinocysts and acritarchs follows Fensome, McRae & Williams (2008).

## Geological Context and Palynological Analysis

The specimen originates from marine deposits of the Breda Formation, which includes Langhian to Gelasian strata (Post & Reumer, 2016).

The preservation and diversity of the dinocysts in the sediment sample taken from NMR 9991-12018 are moderate; many specimens are torn, folded, or broken. A total of 20 dinoflagellate cyst species and one acritarch species were recorded (see Supplemental Information). The biostratigraphical analysis with dinoflagellate cysts and the relative dating of the sediment sample relies on range data from the North Sea Basin and the north Atlantic realm detailed in the publications by De Schepper & Head (2009), Dybkjær & Piasecki (2010) and Louwye, Head & De Schepper (2004).

Although reworking of pre-Neogene dinoflagellate cysts species is considerable, several species have a biostratigraphic value. *Habibacysta tectata* has a lowest occurrence in high latitudes that has been dated at 14.2 Ma by Schreck, Matthiesen & Head (2012), and this datum was later confirmed by Quaijtaal et al. (2014) in lower latitudes (Porcupine Basin, off southwest Ireland). Dybkjær & Piasecki (2010) defined the *Achomosphaera andalousiensis* Zone as the interval from the lowest common occurrence of the eponymous species, recorded in this sample, to the lowest occurrence of *Gramocysta verricula*, and they propose an age of 13.2 Ma for the lower boundary of the zone. *Bitectatodinium serratum* was recorded by Head, Norris & Mudie (1989) for the first time (as *Gongylodinium serratum*) in the upper Miocene of the Labrador Sea. The lowest occurrence of this species aligns with the lower boundary of the Tortonian at 11.6 Ma.

The presence of *Amiculosphaera umbraculum* is noteworthy. The lowest occurrence of this species defines the lower boundary of the eponymous biozone in Denmark, and is placed at 11.4 Ma (early Tortonian) by Dybkjær & Piasecki (2010). The lowest occurrence of *Selenopemphix armageddonensis* defines the lower boundary of the eponymous zone defined by Dybkjær & Piasecki (2010) in Denmark. The latter authors stipulate furthermore that the range of *S. armageddonensis* approximates the range of the zone. This zone has a late Tortonian to earliest Zanclean age (7.6 Ma to 5 Ma), the latter date can provide a minimum age for the sample. *Operculodinium piaseckii* has its highest occurrence within this zone. According to Louwye, Head & De Schepper (2004), *Reticulatosphaera actinocoronata* has a highest occurrence in the North Atlantic realm at 4.4 Ma. Other species recorded in the sample have a younger highest occurrence. *Invertocysta lacrymosa* has a highest persistent occurrence at 2.74 Ma (late Piacenzian) in the eastern North Atlantic realm, while *Operculodinium*? *eirikianum* has its highest occurrence at the upper boundary of the Pliocene, at 2.58 Ma (De Schepper & Head, 2009).

In summary, a tentative relative age between 7.6 Ma and 5 Ma (late Tortonian—earliest Zanclean) can be proposed for this sediment sample.

## Systematic Paleontology

**Table:** 

Order Cetacea Brisson, 1762
Suborder Odontoceti Flower, 1867
Infraorder Delphinida Muizon, 1984
Superfamily Inioidea Gray, 1846 (*sensu* Muizon, 1988a)
Family Pontoporiidae Kasuya, 1973
*Scaldiporia*, gen. nov.

**Type and only included species**: *Scaldiporia vandokkumi*, sp. nov.

**Etymology**: The name of the genus derives from the combination of *Scaldis* (the Roman name of the Westerschelde estuary in which the holotype is found) and *poria*, from the genus name of the only extant member of the family Pontoporiidae, *Pontoporia blainvillei*.

**Diagnosis**: As for the type and only known species.

**Figure 2:**
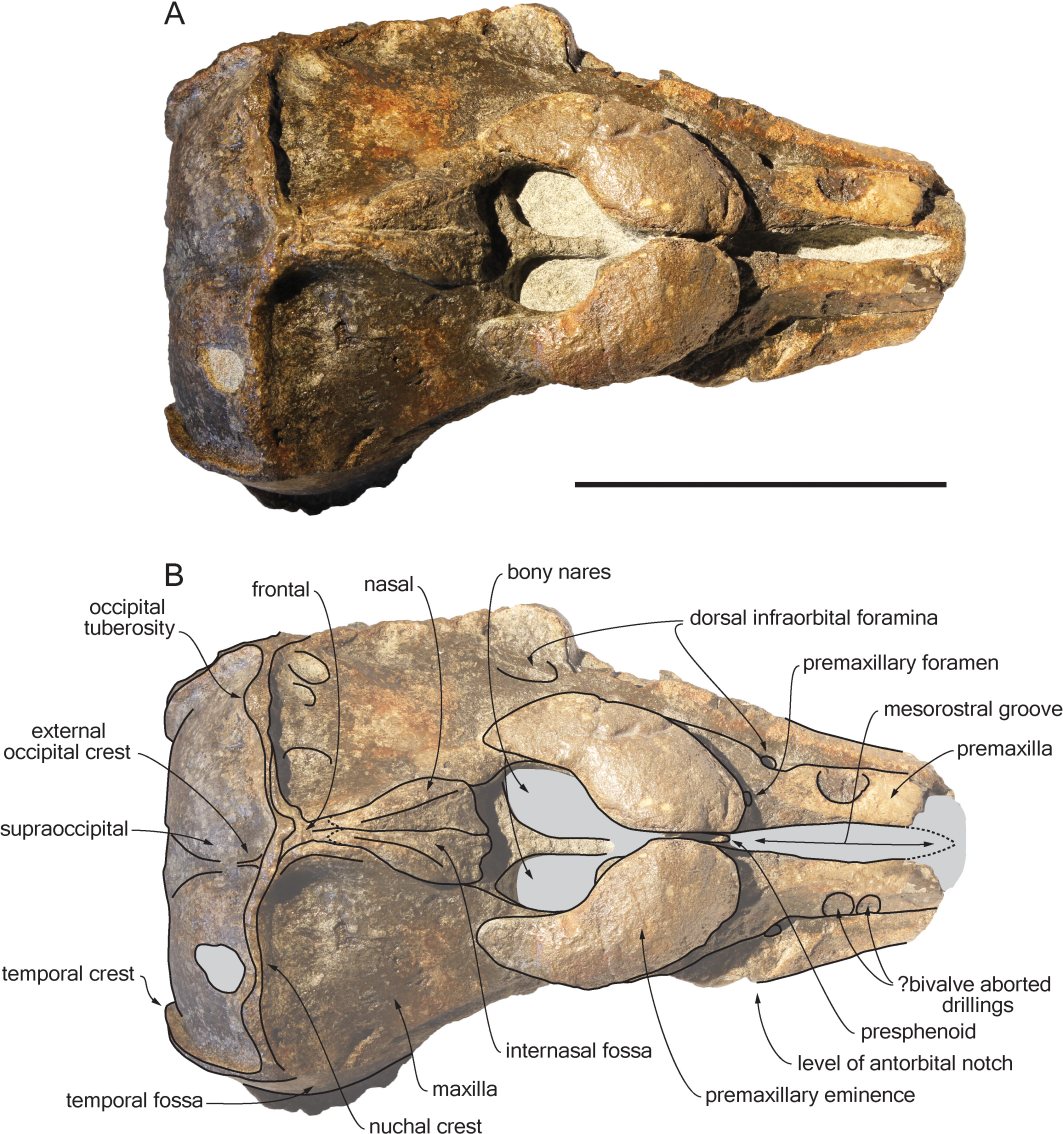
Dorsal view of the cranium of *Scaldiporia vandokkumi*. Photograph (A) and corresponding line drawing (B) of the cranium of the holotype of *S. vandokkumi* NMR 9991-12018 in dorsal view. Grey shading for sediment remaining in cavities. Scale bar equals 100 mm.

**Figure 3:**
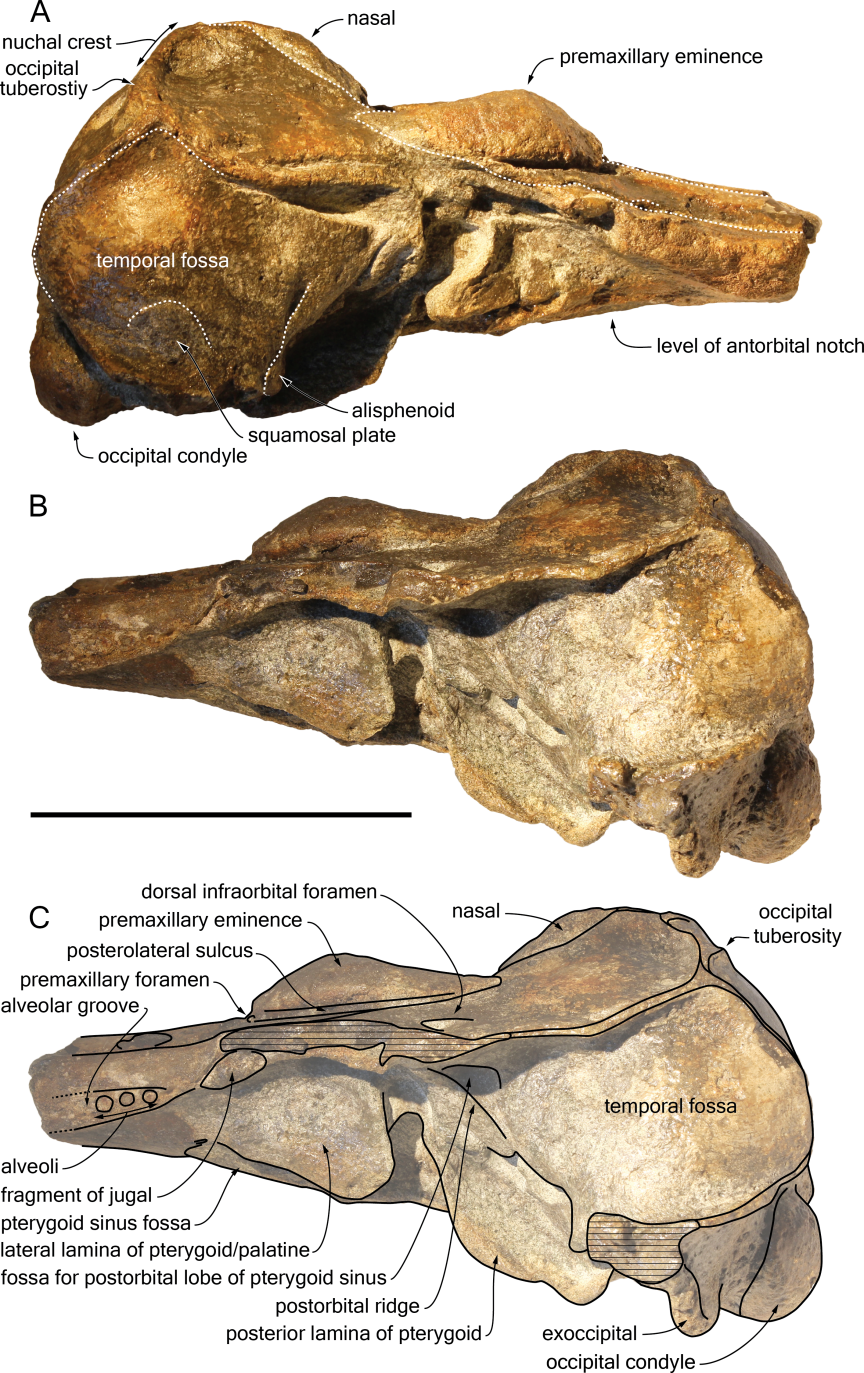
Lateral views of the cranium of *Scaldiporia vandokkumi*. Photograph of the cranium of the holotype of *S. vandokkumi* NMR 9991-12018 in right lateral view (A), photograph in left lateral view (B) and corresponding line drawing (C). Hatching for break surfaces. Scale bar equals 100 mm.

**Figure 4:**
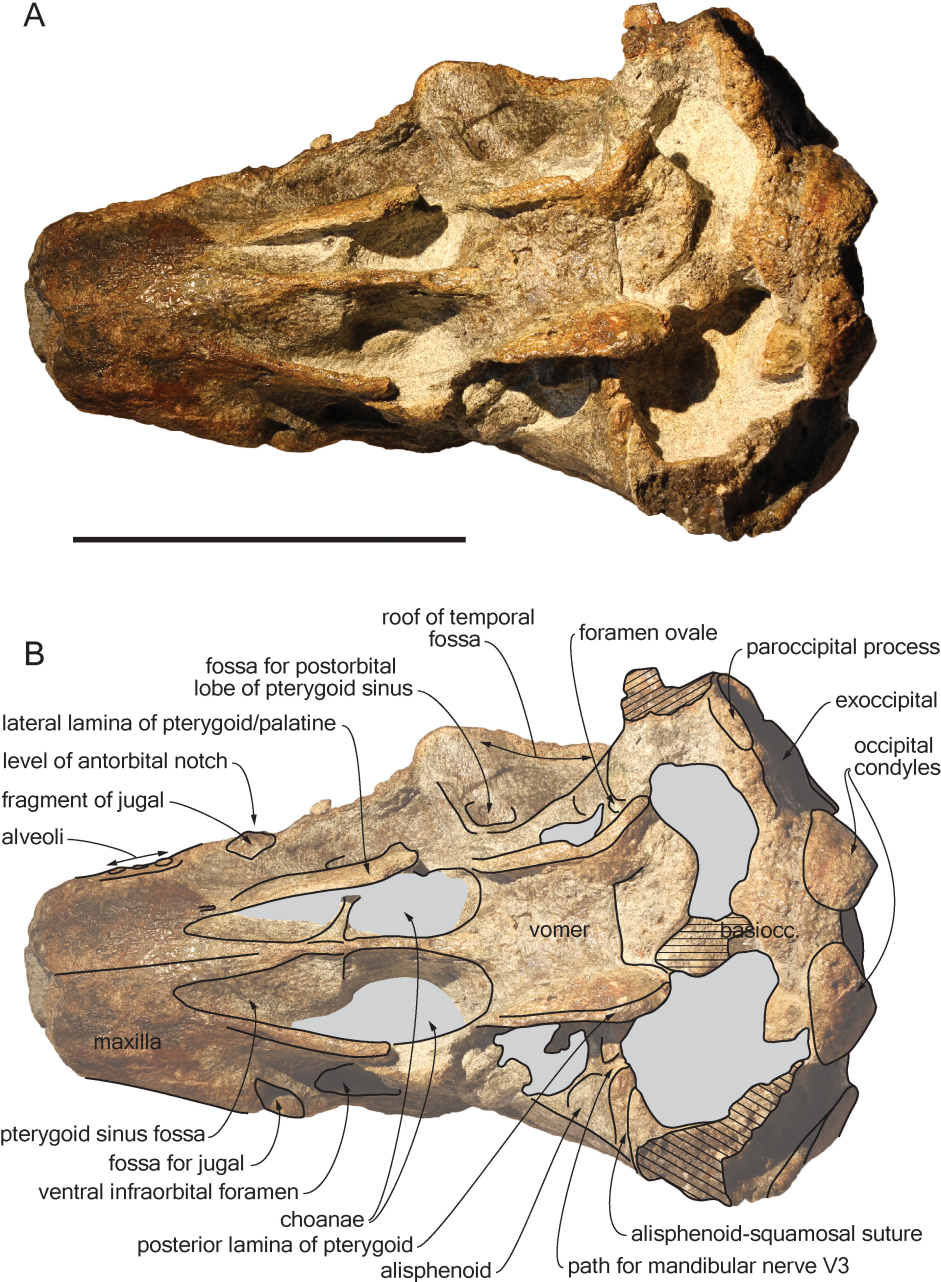
Ventral view of the cranium of *Scaldiporia vandokkumi*. Photograph (A) and corresponding line drawing (B) of the cranium of the holotype of *S. vandokkumi* NMR 9991-12018 in ventral view. Gray shading for sediment remaining in cavities; hatching for break surfaces. Scale bar equals 100 mm.

**Figure 5:**
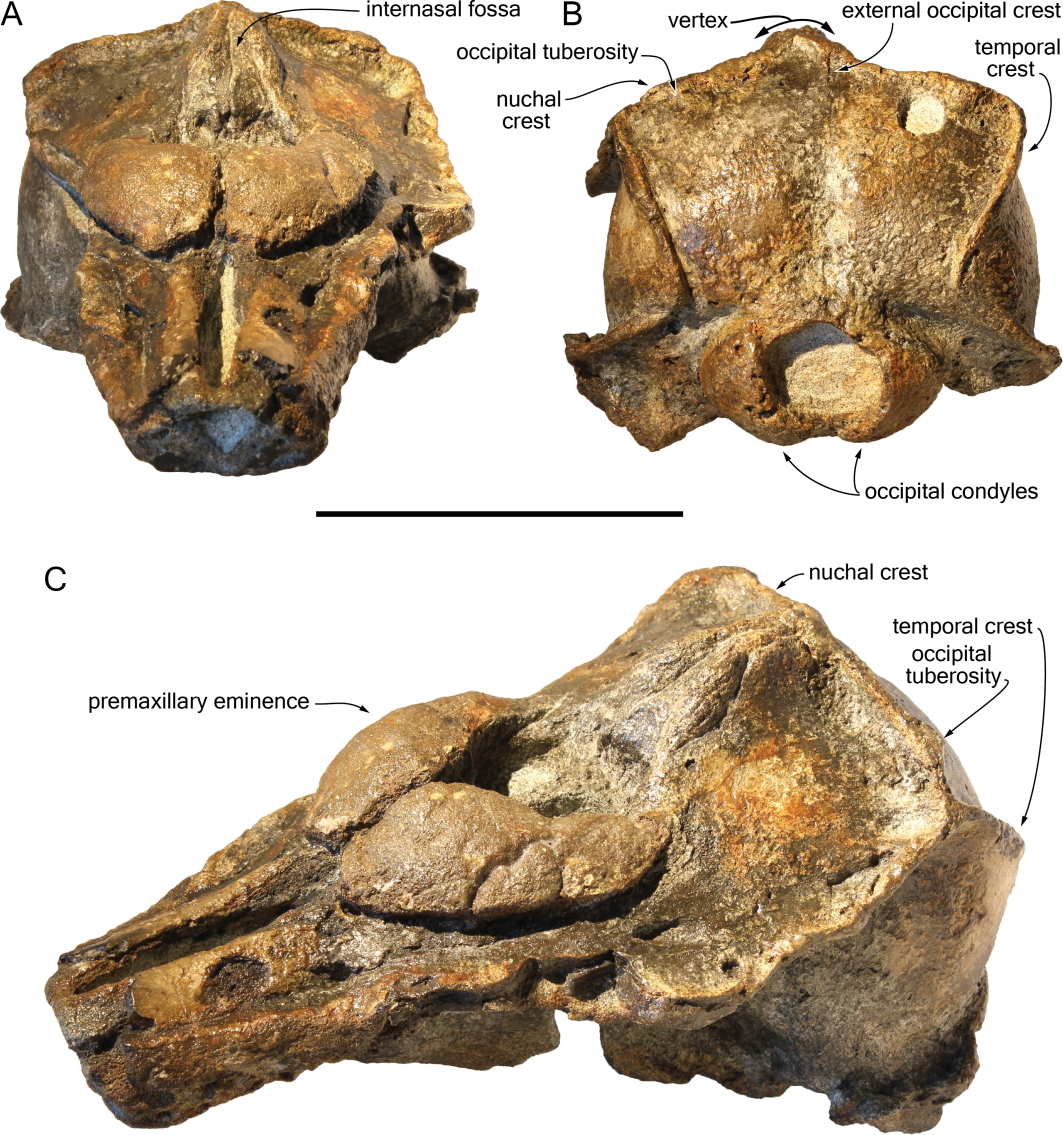
Additional views of the cranium of *Scaldiporia vandokkumi*. Photographs in anterodorsal (A), posterior (B), and left anterodorso lateral (C) view of the cranium of the holotype of *S. vandokkumi* NMR 9991-12018. Scale bar equals 100 mm.

*Scaldiporia vandokkumi*, sp. nov. (Figs. 2–5).

**Holotype**: NMR 9991-12018, a cranium missing apex of rostrum, lateralmost parts of supraorbital regions, part of the squamosals, and petrotympanics. Collected by NMR expedition 2014-3.

**Type locality**: The holotype was recovered in area 6D of the Westerschelde estuary, The Netherlands (Fig. 1), from a depth of 28 metres; geographic coordinates 51°21′569″N–03° 54′251″E (NMR expedition 2014-3, December 17, 2014, tow no 3) (Post & Reumer, 2016).

**Type horizon**: The cranium NMR 9991-12018 originates from the Breda Formation, which includes Langhian to Gelasian strata (Post & Reumer, 2016; Middle Miocene to early Pleistocene). The palynological analysis of sediment from the cerebral cavity of the holotype suggests an age between 7.6 and 5.0 Ma (late Tortonian to earliest Zanclean, Late Miocene to earliest Pliocene; see above and Supplemental Information). From the same site and same lithological unit, an unidentified odontocete, a large ziphiid, a relatively small balaenopteroid, two different cetotheres, a large leatherback turtle, and a very large shark were recovered (Post & Reumer, 2016). Future research will decide whether or not they are from the same age and fauna as NMR 9999-12018. The sediment of NMR 9991-12018 contained but one single mollusc (Lucinidae, cf. *Lucinoma borealis*, paired valves). This bivalve bears no specific stratigraphic information (being present in the North Sea realm from the Eocene onwards), but indicates (often) a neritic, anaerobic environment (Taylor & Glover, 2000).

**Etymology**: The species name honours captain Jan van Dokkum, one of the few captains daring to brave the dangerous sediments, currents and commercial traffic of the Westerschelde area in search of fossil marine mammals.

**Diagnosis of species**: With a bizygomatic width greater than 14 cm, the robust cranium of *Scaldiporia vandokkumi* differs from other inioids in the autapomorphic presence of large, dorsoventrally thick (23 mm) premaxillary eminences reaching the level of the antorbital notch, in both the posteromedial and posterolateral sulci being consequently not dorsally visible, and in the presence of occipital tuberosities on the posterior surface of the nuchal crest. It further differs from other inioids in the following unique combination of characters: dorsomedially sloping dorsal surface of the premaxilla at the rostrum base; maxilla narrower than premaxilla at rostrum base; progressive (versus abrupt) elevation of the dorsal surface of the maxilla from rostrum base towards antorbital region; presphenoid not present between premaxillae anterior to premaxillary eminences; lateral edge of bony nares formed by maxilla posteriorly; posterior apices of premaxillae not diverging; posterior edge of the premaxilla distant from the nasal; right premaxilla roughly reaching the anteroposterior level of the posterior margin of the bony nares; poorly elevated vertex; vertex distinctly shifted to the left side of the asymmetric cranium; anteroposteriorly elongated nasals; nasals posteriorly pointed; deep internasal fossa; supraoccipital barely anteriorly wedged between frontals and maxillae, with nuchal crest nearly straight; supraoccipital shield with distinct trapezoid outline in posterior view, due to posteromedial extension of the temporal fossa; and presence of a fossa for the postorbital lobe of the pterygoid sinus.

## Description

**General features**: Although the dorsal, posterior, and lateral surface of the neurocranium of NMR 9991-12018 are finely preserved, large gaps appear between the condylar region, the squamosals, and the anterior part of the basicranium, revealing wide ventral openings of the cerebral cavity. However, a partly abraded longitudinal bony bridge between the posterior and anterior parts of the basicranium suggests that no major break and shift occurred during burial/fossilization. With a bizygomatic width greater than 140 mm (Table 1), this cranium is broader than the skull of *Pontoporia blainvillei* (see Muizon, 1984, Table 1), closer to *Pliopontos littoralis*. The lateral margins of the rostrum at its base are abraded; no estimate of the rostrum length could be provided. The moderately elevated vertex region is shifted to the left side as compared to the sagittal groove on the supraoccipital shield, revealing a degree of asymmetry greater than in *Pontoporia* (having a symmetric to slightly asymmetric cranium; Gutstein et al., 2009). The most striking character of the cranium is the robust, dome-like premaxillary eminences, proportionally larger than in any other known odontocete. The temporal fossa is high and long, extending far posteromedially.

**Table 1:** Measurements (in mm) of the cranium of *Scaldiporia vandokkumi* NMR 9991-12018 (holotype).

	NMR 9991-12018
Total length of cranium as preserved	212
Rostrum length as preserved	53
Neurocranium length	159
Maximum dorsal opening of mesorostral groove	9.5
Width of rostrum at base	e72
Width of premaxillae at rostrum base	48
Maximum width of premaxillary eminences	76
Maximum width of right premaxillary eminence	33
Maximum width of left premaxillary eminence	32
Width of bony nares	37
Maximum width of nasals	26
Minimum distance between maxillae across vertex	5.5
Postorbital width of cranium	+132
Bizygomatic width of cranium	+140
Distance between lateral margins of temporal crests across nuchal crest in dorsal view	111
Minimum distance between medial margins of temporal crests across occipital shield in posterior view	69
Maximum height of right temporal fossa (to floor of squamosal fossa)	65
Maximum height of left temporal fossa (to floor of squamosal fossa)	62
Height of vertex above top of temporal fossa	29
Width of occipital condyles	63.5
Width of right occipital condyle	21
Width of left occipital condyle	20
Height of right occipital condyle	+32.5
Height of left occipital condyle	36
Width of foramen magnum	31
Height of foramen magnum	27.5
Maximum width across exoccipitals	e134

**Notes.**

e estimate + incomplete

**Ontogenetic stage**: Taking account of the general robustness of the bones, the strong nuchal and temporal crests, the degree of fusion of the cranial sutures, and the well-defined posterior maxillary alveoli, the specimen NMR 9991-12018 is considered as fully adult.

**Premaxilla**: Traces of both premaxillae left on sediment at the preserved transverse section of the rostrum indicate that the anterior parts of the right and left premaxillae were contacting each other over the mesorostral groove. Prudent approach prevents this observation to be included in the diagnosis. More posterior (on the right premaxilla after 7 mm, on the left premaxilla after 14 mm) the premaxillae are well preserved and are separated until the level of the premaxillary eminences, leaving the wide and deep mesorostral groove open dorsally (Fig. 2). In this area the dorsal surface of the premaxilla slopes dorsomedially with an angle of ca. 35% with the horizontal. The porcelanous surface of the premaxillae shows circular depressions (two on the right—, and one on the left premaxilla). Similar depressions were also noted in the dorsal surface of the compact rostral and facial bones of the ziphiid *Ziphirostrum marginatum* from the Miocene of Belgium, and considered to be aborted drillings by bivalves (Lambert, 2005). The premaxilla widens to 55 mm, before developing into a wide and bulbous premaxillary eminence with a markedly transversely and longitudinally convex dorsal surface and a maximum thickness of 23 mm (taken from the anterolateral contact with the maxilla) anterior to the bony nares (Figs. 2, 3 and 5). More voluminous than in any other inioid (taking account of the intraspecific variation in extant species) and roughly reaching anteriorly the level of the antorbital notch, this eminence at least partly overhangs the premaxillary foramen anteriorly (the latter being only detected on the left side; Figs. 2, 5A, 5C) and the posterolateral sulcus laterally. The relatively spongy aspect of the surface and minor breaks in the eminences indicate a bone distinctly less compact than the anterior porcelanous portion of the premaxilla. No posteromedial sulcus could be detected, and due to the slightly damaged medial margin of each premaxilla, no anteromedial sulcus and prenarial triangle could be described. Only *Auroracetus bakerae* Gibson & Geisler, 2009, and *Awadelphis hirayamai* Murakami, 2016 show—to a lesser extent—some of these features (relatively high premaxillary eminence and posteromedial sulcus dorsally not visible). However, the eminence of *A. bakerae* is sloping anteromedially into a concavity, and is not overhanging the premaxilla and part of the maxilla laterally. In *A. hirayamai* the anterior half of the eminence is unfortunately not preserved. Finally, it is impossible to assess if the eminence was originally reaching the level of the antorbital notch in specimens of both taxa. The eminence extends posterolaterally on the sides of the bony nares, while gradually decreasing in height and width. The moderately tapering posterior end of the premaxilla diverges from the bony nares and its broad apex remains distant from the corresponding nasal (10.5 mm on the right side and 12 mm on the left). The left eminence is not completely preserved posterolaterally, but the maxilla still bears the traces of the maxilla-premaxilla suture, and therefore marks its broad original outline.

**Maxilla**: The transverse section of the broken apex of the rostrum shows firmly sutured, triangular shaped maxillae, each of them being pierced by a single canal, presumably the incisivomaxillary canal. Dorsally the maxilla is as wide as the premaxilla at the preserved apex, from where it gradually widens and raises towards the antorbital notch. In lateral view, at the preserved apex of the rostrum the lateral margin of the maxilla makes a 23 mm thick bone mass, which gradually decreases in thickness to 14 mm at the location of the antorbital notch (Fig. 3). From the anterior end to ca. 12 mm before the antorbital notch, a shallow but wide alveolar groove is partly preserved on the left side, displaying faint remains of at least four oval shaped alveoli (Figs. 3 and 4). Slightly anterior to the level of the right premaxillary foramen and at about the level of the lost antorbital notch, a small dorsal infraorbital foramen pierces each maxilla along the suture with the premaxilla. The maxilla continues alongside the premaxillary eminence as a wide, subhorizontal, thin layer of bone over the lacrimal and supraorbital process of the frontal. The bone is unfortunately too incomplete in the supraorbital region for detecting the presence of a longitudinal maxillary crest. Between the level of the lost postorbital process and the posterior part of the premaxillary eminence, a second dorsal infraorbital foramen is present at a short distance from the overhanging lateral margin of the premaxillary eminence. Beyond the orbital region, the maxilla raises posterodorsally until it reaches the anterior wall of the robust and elevated nuchal crest. Along the lateral wall of the vertex the maxilla is markedly dorsomedially elevated, laterally bordering the nasal and the limited exposure of the frontal on the vertex. The dorsomedial elevation is much more abrupt on the right side, making a subvertical wall contrasting with the lower slope of the left maxilla. The posterolateral edge of the left maxilla raises slightly dorsolaterally, a condition reminiscent of the elevated edge observed for example in *Pontoporia*.

**Presphenoid**: The anterior border of the ossified presphenoid (*sensu* (Ichishima, 2016)) is located between the high anteromedial walls of the premaxillary eminences (Figs. 2, 5A). More posterior, a low, ca. 4 mm thick nasal septum separates the bony nares, widening to a ca. 30 mm wide transverse posterior plate reaching dorsally the anteroventral margin of the nasals. In this area, the presphenoid is made of more cancellous bone compared to the other bones of the cranium, and the superficial layer of this fragile bone may have been lost during preparation.

**Nasal**: The nasal is shaped like an elongated posterodorsally pointed triangle, which develops—over about 45 mm—from a 12 mm wide anterior base at the contact with the cribriform plate, into a 1 mm pointed posterior edge contacting the exposure of the frontal on the vertex (Fig. 2). This outline is more similar to the Late Miocene *Pontistes* than to any other pontoporiid. The anterior margin of each nasal is cut by a shallow vertical notch, as seen in *Pontoporia*. The dorsomedial surface of the joined nasals is excavated by a deep internasal fossa, separating two bulging regions converging posteriorly (Figs. 2, 5A, 5C). The posterior part of the bulging region slightly overhangs the maxilla.

**Frontal**: The left supraorbital process of the frontal extends for at least 30 mm laterally at the level of the lost postorbital process allowing the estimation of a minimum postorbital width. More anteriorly, only vertical sections of the frontal are visible under the thin maxilla covering the antorbital region. A deep fossa for the postorbital lobe of the pterygoid sinus is observed posterior to the left infratemporal crest (Fig. 4), as in *Brachydelphis* but not *Pontoporia* (Fraser & Purves, 1960; Gutstein et al., 2009; Lambert & Muizon, 2013). In lateral view, the suture of the anteriormost part of frontal with the corresponding maxilla in the area of the antorbital notch is feebly S shaped. On the vertex the frontals are visible as anteroposteriorly elongated, narrow, and transversely slightly convex stripes of bone between the dorsomedially elevated maxillae.

**Lacrimal**: The lacrimal is eroded/absent for most part. Nevertheless, the lack of an abrupt posterodorsal elevation of the maxilla in the antorbital notch region suggests that no swollen, dorsoventrally thick lacrimal/maxilla complex was present in the antorbital region, differing thus from the condition typical for *Brachydelphis*. However, this area is too damaged to consider this observation as final.

**Jugal**: Medial to the left antorbital notch, a small rounded bony structure embedded in the ventral surface of the maxilla most likely represents the base of the styliform process of the jugal (Figs. 3 and 4). On the right side of the cranium, a triangular depression in the maxilla corroborates this observation.

**Supraoccipital**: Apart from a small circular cavity in the right upper part, the supraoccipital is completely preserved. In posterior view, the supraoccipital shield displays a general trapezoid outline, with the largest side formed by the nuchal crest and the lateral sides formed by the ventromedially converging temporal crests (Fig. 5B). The prominent nuchal crest is nearly straight in dorsal view, only sending a short and narrow anteromedial wedge between the maxillae (Fig. 2). The highest point of the nuchal crest is located somewhat to the left side of the sagittal plane, confirming the asymmetry of the cranium (Fig. 5B). Furthermore, the left side of the crest slightly overhangs the posterior margin of the maxilla, which is not the case on the right side. Exactly in the middle of the nuchal crest a tiny vertically directed external occipital crest is present for over 20 mm. On the posterior wall of the nuchal crest, ca. 20 mm from its lateral side, an occipital tuberosity most likely corresponds to neck muscle attachment (see discussion below; Figs. 2 and 3, 5B). The temporal crest is robust and prominent over its entire length, projecting posteromedially. In lateral view the supraoccipital shield shows a generally convex outline, sloping from the nuchal crest anterodorsally to the condyles posteroventrally. A slight sagittal depression runs from the external occipital crest to the top of the foramen magnum, defining externally the cerebral hemispheres.

**Exoccipital**: The left exoccipital is almost complete and its concave posterior surface widens ventrolaterally towards the squamosal (Fig. 5B). The robust occipital condyles border a relatively high and wide foramen magnum and are separated by a 6 mm wide intercondyloid notch.

**Basioccipital**: As mentioned above parts of the basioccipital and surrounding bones are missing. This includes the posterior part of the transversely thin basioccipital crests and part of the floor of the basioccipital basin. The anterior part of the left crest curves posterolaterally in a way that indicates some degree of diagenetic distortion (Fig. 4).

**Vomer**: The floor of the basioccipital basin is cut by a transverse step most likely corresponding to the posterior margin of the vomer. Anteriorly the vomer gradually becomes keeled and contributes ventrally to the nasal septum.

**Parietal**: On the medial wall of the anteroposteriorly long temporal fossa, the parietal extends posteriorly until a level in line with the occipital condyles. In posterior view, the surface of the parietal in the fossa is slightly laterally convex, while the posterior border curves inwards over the supraoccipital shield (Fig. 5B).

**Squamosal**: Parts of the suture between the squamosal plate and the parietal are visible in the right temporal fossa (Fig. 3A). The transversely concave floor of the narrow squamosal fossa and the proximal part of the postglenoid process are partly preserved on both sides..

**Palatine**: The anteriormost position of the palatine is difficult to determine because a clear maxilla-palatine suture could not be identified. Nevertheless, considering the anterior extent of the pterygoid sinus fossa (Fig. 4), the palatine extended on the transversely convex ventral surface of the rostrum for more than 30 mm. The lateral lamina of the palatine reaches posteriorly at least until mid-length of the choana. However, no suture could be detected along the dorsoventrally high lamina laterally defining the pterygoid sinus fossa; pterygoid and palatine contributions could thus not be assessed.

**Pterygoid**: The ventral surface of the hamular process is missing, and therefore the anteriorly directed fairly narrow pterygoid sinus fossa of which the medial lamina and the lateral lamina are preserved is ventrally exposed, extending beyond the base of the rostrum. Ventrolateral to the vomer, the thin posterior lamina of the pterygoid defines laterally the anterior part of the basioccipital basin. The lateral surface of this lamina deepens markedly dorsomedially, which suggests a voluminous pterygoid sinus fossa in this region. The pterygoid-basioccipital suture could not be detected.

**Alisphenoid**: Only the anterior wall of the foramen ovale is preserved on both sides, followed anterolaterally towards the temporal fossa by the path for the mandibular nerve V3 (Fig. 4). Just anterior to this path, the ventral surface of the alisphenoid is anteroposteriorly concave.

## Comparison

**Figure 6:**
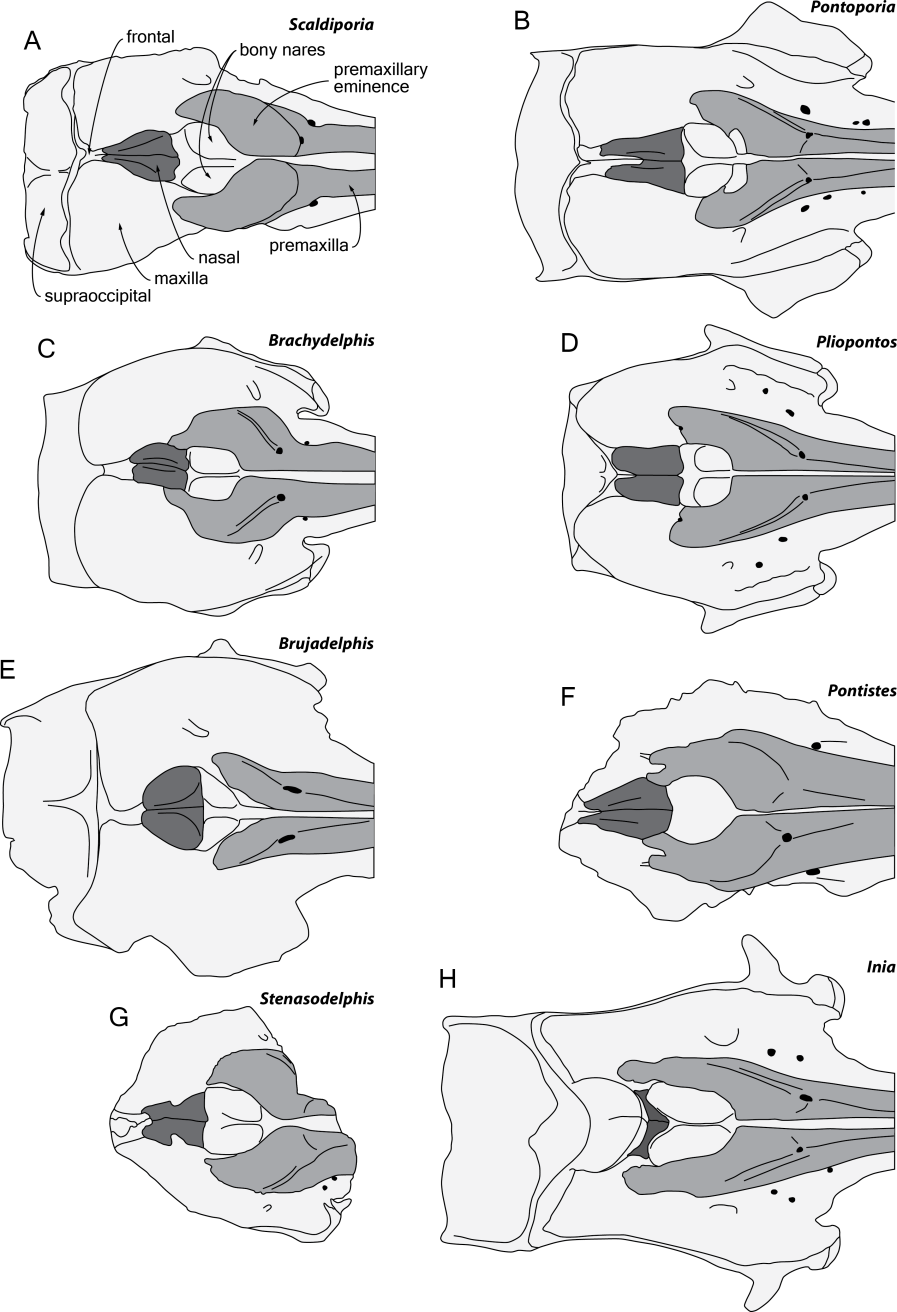
Comparison of the neurocranium of *Scaldiporia vandokkumi* with other inioids in dorsal view. Schematic line drawings of: (A) *S. vandokkumi*; (B) *Pontoporia blainvillei*, based on ZMA 16.714; (C) *Brachydelphis mazeasi*, modified from Muizon (1988b); (D) *Pliopontos littoralis*, modified from Muizon (1984); (E) *Brujadelphis ankylorostris*, modified from Lambert et al. (2017); (F) *Pontistes rectifrons*, based on the holotype MACN 3190; (G) *Stenasodelphis russellae*, modified from Godfrey & Barnes (2008); (H) *Inia geoffrensis*, based on ZMA 17.771. All specimens reduced to the same maximum width of the premaxillary sac fossae, except for *B. ankylorostris*. Light grey shading for premaxillae and dark grey shading for nasals.

The content and definition of the two inioid families Iniidae and Pontoporiidae is still subject to debate, with intermediary forms identified and with different phylogenetic analyses producing contrasted results (Cozzuol, 2010; Geisler, Godfrey & Lambert, 2012; Pyenson et al., 2015; Murakami, 2016; Aguirre-Fernández et al., 2017; Lambert et al., 2017). Nevertheless, in the absence of ear bones the combination of characters placing the new taxon in the family Pontoporiidae is: (1) markedly swollen premaxillary eminences, with a transversely convex dorsal surface (also present to some extent among iniids and phocoenids); (2) greatly anteroposteriorly elongated nasals; (3) presence of an internasal fossa; and (4) poorly elevated vertex (see Muizon, 1984; Muizon, 1988a; Muizon, 1988b; Lambert & Muizon, 2013). The three last characters contrast with the extant iniid *Inia* (Fig. 6) and, in part, with some fossil relatives (*Awadelphis*, *Brujadelphis*, *Ischyrorhynchus*, *Isthminia*, and *Meherrinia*).

Among pontoporiids, *S. vandokkumi* differs from:

***Protophocaena minima*** (?Middle to Late Miocene of Belgium and The Netherlands; Lambert & Post, 2005) in: larger size; presphenoid not present between premaxillae anterior to premaxillary eminences; larger and dorsoventrally thicker premaxillary eminence; posteromedial and posterolateral sulci not dorsally visible (related to the great development of the premaxillary eminences); lateral edge of bony nares formed by maxilla posteriorly; posterior edge of the premaxilla distant from the nasal; and subhorizontal surface of the maxilla lateral to the posterior dorsal infraorbital foramen (versus ventrolateral slope).

**MGUH 1910-274** (Pontoporiidae gen. et sp. indet.; Tortonian of Denmark; Pyenson & Hoch, 2007) in: dorsomedially sloping dorsal surface of the premaxilla at the rostrum base (versus ventromedially); larger, dorsoventrally thicker premaxillary eminence reaching anteriorly the level of the antorbital notch; posteromedial and posterolateral sulci not dorsally visible; posterior edge of the premaxilla distant from the nasal; progressive (versus abrupt) elevation of the dorsal surface of the maxilla from rostrum base towards antorbital region (condition in MGUH 1910-274 is associated to dorsoventrally thick maxilla and lacrimal in the antorbital region); thick maxillary crest of maxilla over the supraorbital process of the frontal most likely absent; and maxilla subhorizontal in the posteromedial part of the orbit region.

**MGUH 1922-168** (cf. *Pontiste*s sp.; Tortonian of Denmark; Pyenson & Hoch, 2007) in: larger, dorsoventrally thicker premaxillary eminence reaching anteriorly the level of the antorbital notch; posteromedial and posterolateral sulci not dorsally visible; lateral edge of bony nares formed by maxilla posteriorly; thick maxillary crest of maxilla over the supraorbital process of the frontal most likely absent; nasals posteriorly pointed; deep internasal fossa; and maxilla subhorizontal in the posteromedial part of the orbit region.

***Auroracetus bakerae*** (Zanclean of North Carolina, USA; Gibson & Geisler, 2009) in: larger, dorsoventrally thicker premaxillary eminence reaching anteriorly the level of the antorbital notch; posterolateral sulci not dorsally visible; right premaxilla roughly reaching the level of the posterior margin of the bony nares (much shorter in *Auroracetus*); and nasals posteriorly pointed.

***Stenasodelphis russellae*** (Tortonian of Maryland, USA; Godfrey & Barnes, 2008; Fig. 6G) in: presphenoid not present between premaxillae anterior to premaxillary eminences; larger, dorsoventrally thicker premaxillary eminence reaching anteriorly the level of the antorbital notch; posteromedial and posterolateral sulci not dorsally visible; right premaxilla roughly reaching the level of the posterior margin of the bony nares (much shorter in *Stenasodelphis*); posterior dorsal infraorbital foramen posterior to anterior margin of bony nares; deep internasal fossa.

***Brachydelphis mazeasi*** **and** ***Brachydelphis jahuayensis*** (?Middle to Late Miocene of Chile and Peru; Muizon, 1984; Muizon, 1988b; Gutstein et al., 2009; Lambert & Muizon, 2013; Bianucci et al., 2016a; Bianucci et al., 2016b; Di Celma et al., 2017; Fig. 6C) in: presphenoid not present between premaxillae anterior to premaxillary eminences; larger, dorsoventrally thicker premaxillary eminence reaching anteriorly the level of the antorbital notch; posteromedial and posterolateral sulci not dorsally visible; posterior edge of the right premaxilla distant from the nasal; progressive (versus abrupt) elevation of the dorsal surface of the maxilla from rostrum base towards antorbital region; more posteriorly pointed nasals; deep internasal fossa; thick maxillary crest of maxilla over the supraorbital process of the frontal most likely absent; maxilla subhorizontal in the posteromedial part of the orbit region; presence of occipital tuberosities on posterior surface of the nuchal crest; and supraoccipital shield with distinct trapezoid outline in posterior view, due to posteromedial extension of the temporal fossa.

***Pontistes rectifrons*** (Late Miocene of Argentina; Cozzuol, 2010; Fig. 6F) in: presphenoid not present between premaxillae anterior to premaxillary eminences; larger, dorsoventrally thicker premaxillary eminence reaching anteriorly the level of the antorbital notch; posteromedial and posterolateral sulci not dorsally visible; lateral edge of bony nares formed by maxilla posteriorly; and posterior edge of the premaxilla distant from the nasal.

***Pliopontos littoralis*** (Messinian to ?Zanclean of Peru; Muizon, 1984; Ehret et al., 2012; Fig. 6D) in: larger, dorsoventrally thicker premaxillary eminence reaching anteriorly the level of the antorbital notch; posteromedial and posterolateral sulci not dorsally visible; posterior apices of premaxillae not diverging; no foramen along the posterior margin of the premaxilla; posteriorly pointed nasals; deep internasal fossa; supraoccipital much less anteriorly wedged between frontals and maxillae, with nuchal crest nearly straight; presence of occipital tuberosities on posterior surface of the nuchal crest; and supraoccipital shield with distinct trapezoid outline in posterior view, due to posteromedial extension of the temporal fossa.

***Pontoporia blainvillei*** (Recent, east coast of South America; Fig. 6B) in: larger, dorsoventrally thicker premaxillary eminence reaching anteriorly the level of the antorbital notch; posteromedial and posterolateral sulci not dorsally visible; posterior apices of premaxillae not diverging; proportionally higher vertex; asymmetric vertex, distinctly shifted to the left; deep internasal fossa; presence of occipital tuberosities on posterior surface of the nuchal crest; supraoccipital shield with distinct trapezoid outline in posterior view, due to posteromedial extension of the temporal fossa; and presence of a fossa for the postorbital lobe of the pterygoid sinus.

Among non-pontoporiid inioids sharing some similarities at the level of the facial region with *S. vandokkumi*, the latter differs from:

***Awadelphis hirayamai*** (Messinian of Japan; Murakami, 2016) in: larger, dorsoventrally thicker premaxillary eminence; posterior edge of premaxilla distant from nasal; supraoccipital much less anteriorly wedged between frontals and maxillae, with nuchal crest nearly straight; and presence of occipital tuberosities on posterior surface of nuchal crest.

***Isthminia panamensis*** (Messinian of Panama; Pyenson et al., 2015) in: mesorostral groove dorsally open at rostrum base; large, dorsoventrally thick premaxillary eminence reaching the level of the antorbital notch; maxilla narrower than premaxilla at rostrum base; much smaller dorsal infraorbital foramen at rostrum base; less elevated vertex (compared to dorsal margin of rostrum); deep internasal fossa; squared posteriolateral border of maxilla square (versus rounded); presence of occipital tuberosities on posterior surface of nuchal crest; and transversely broader upper part of supraoccipital shield.

***Brujadelphis ankylorostris*** (Serravallian to early Tortonian of Peru; Lambert et al., 2017; Fig. 6E) in: larger, dorsoventrally thicker premaxillary eminence reaching the level of the antorbital notch; posteromedial and posterolateral sulci not dorsally visible; maxilla narrower than premaxilla at rostrum base; less elevated vertex (compared to dorsal margin of rostrum); less dorsally inflated nasals; proportionally anteroposteriorly longer nasals; presence of occipital tuberosities on posterior surface of nuchal crest; and supraoccipital shield with distinct trapezoid outline in posterior view, due to posteromedial extension of the temporal fossa.

***Meherrinia isoni*** (Messinian of North Carolina, USA; Geisler, Godfrey & Lambert, 2012) in: presphenoid not present between premaxillae anterior to premaxillary eminences; large, dorsoventrally thick premaxillary eminence reaching the level of the antorbital notch; posterolateral sulcus not dorsally visible; less elevated vertex; proportionally anteroposteriorly longer nasals; posteriorly pointed nasals; deep internasal fossa; presence of occipital tuberosities on posterior surface of nuchal crest; and supraoccipital less anteriorly wedged between frontals and maxillae, with nuchal crest nearly straight.

## Phylogenetic Relationships

Preliminary phylogenetic analyses including all the OTU’s from Lambert et al. (2017) resulted in highly volatile relationships for several fragmentarily known extinct inioids and early delphinidans (e.g., *Auroracetus bakerae*, *Ischyrorhynchus vanbenedeni*, *Pithanodelphis cornutus*, and *Protophocaena minima*). Unsurprisingly, the lack of information on ear bones generates much less robust relationships, and as our primary goal is not to resolve relationships among early branching delphinidans, but rather to test the pontoporiid affinities of *Scaldiporia vandokkumi*, we removed from our dataset seven OTU’s based on relatively fragmentary material (matrix in supplementary material). The phylogenetic analysis yielded two most parsimonious trees with a score of 1620.96668 steps, a consistency index (CI) of 0.16, and a retention index (RI) of 0.57.

**Figure 7:**
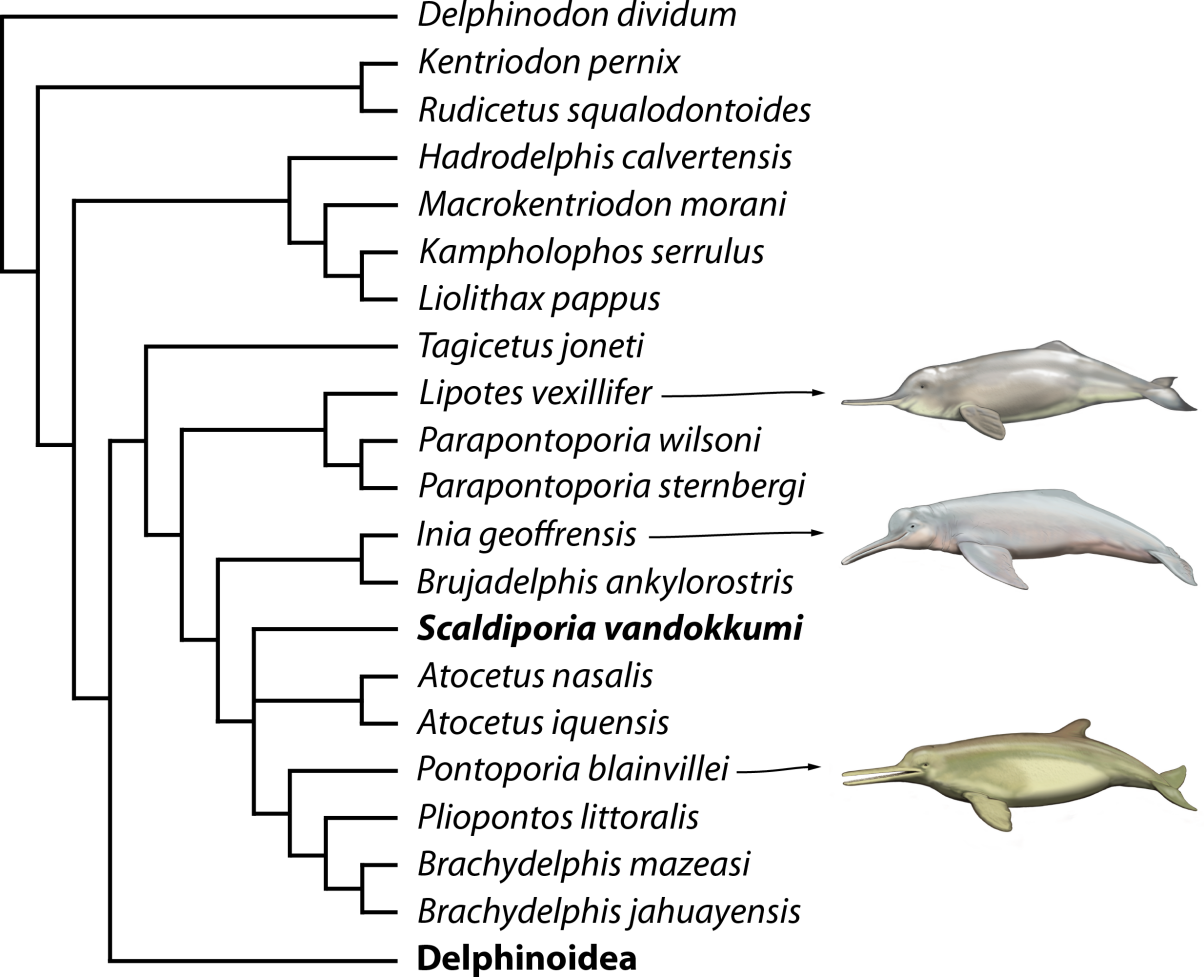
Phylogenetic relationships of *Scaldiporia vandokkumi* with other inioids. Result of our phylogenetic analysis based on the matrix of Lambert et al. (2017). Heuristic search with a backbone molecular constraint resulted in two most parsimonious trees whose strict consensus is provided as supplementary material. Only relationships among inioids and other early delphinidans are shown here, with *S. vandokkumi* being more closely related to *Pontoporia blainvillei* than to *Inia geoffrensis*. Life reconstructions of extant *I. geoffrensis*, *Lipotes vexillifer*, and *P. blainvillei* by C Buell.

Only the part of the strict consensus tree dealing with inioids and other early branching delphinidans is shown in Fig. 7 (complete tree in Supplemental Information). As demonstrated in the analyses of Lambert et al. (2017), a series of Miocene delphinidans (generally named ‘kentriodontids’) diverge before Lipotidae + Inioidea. Among inioids, *S. vandokkumi* is more closely related to *Pontoporia blainvillei* than to *Inia geoffrensis*, a result confirming our attribution of this new taxon to the family Pontoporiidae. However, we find a somewhat unexpected position of *Atocetus iquensis* and *Atocetus nasalis* among pontoporiids. Considering the removal of a series of inioids from our dataset due to the highly fragmentary state of the specimens on which they are based, we refrain from commenting more in detail on relationships within the superfamily, pending the discovery of more complete specimens for *Auroracetus bakerae*, *Awadelphis hirayamai*, *Ischyrorhynchus vanbenedeni*, *Meherrinia isoni*, *Protophocaena minima*, and *Stenasodelphis russellae*).

## Discussion

**Morphological features**: Together with some other inioids and phocoenids, all known pontoporiid taxa show premaxillary sac fossae which are slightly to moderately upheaved, forming more or less prominent premaxillary eminences bordered by posteromedial and posterolateral sulci (the latter features being weakly preserved or absent in *Auroracetus* and *Awadelphis*: Gibson & Geisler, 2009; Murakami, 2016). However, none of the currently described pontoporiids presents this feature as thick and extended as in the holotype of the new genus and species *Scaldiporia vandokkumi*, in which the sulci are completely covered or overhung by the eminence. Differing from the massive prominences occurring on the rostrum of several fossil ziphiids (e.g., Bianucci et al., 2013), the premaxillary eminences of *S. vandokkumi* are not made of compact bone. Furthermore, they are located more posteriorly in the facial region, possibly under premaxillary sacs. Described as the smallest diverticulae in *Pontoporia* (Frainer, Huggenberger & Moreno, 2015), but also observed in many non-physeteroid extant odontocetes, the latter are air sacs most likely involved, together with other forehead structures (nasal plugs, other nasal diverticulae, phonic lips) in the production of echolocation sounds (Mead, 1975; Heyning, 1989; Cranford, Amundin & Norris, 1996; Cranford et al., 2011; Cranford et al., 2014). Modifications of the bony architecture of this region may indicate changes in the morphology/function of the overlying soft tissue structures. Interestingly, no change in size and shape of the premaxillary sacs was detected between neonates and adults of *Pontoporia* (Frainer, Huggenberger & Moreno, 2015).

Another interesting feature of *S. vandokkumi* is the presence of a pair of occipital tuberosities located along the posterior surface of the nuchal crest. Those are interpreted as corresponding to the origin of axial or neck muscles, either M. semispinalis capitis or rectus capitis posterior major; the former fuses with M. longissimus in the thoracic region of *Pontoporia*, whereas the latter inserts on the axis, and both are involved in the lateral flexion/extension of the head (see Schulte, Smith & Andrews 1918; Strickler, 1980; Pabst, 1990). Even more developed occipital tuberosities are observed in the gray whale and relatives (e.g., Ichishima et al., 2006).

More generally, the robustness of the cranium of the holotype of *S. vandokkumi*, its relatively high nuchal and temporal crests, and its greatly thickened premaxillary eminences contrast markedly with the slender skull of extant *Pontoporia blainvillei*. Although such differences may tentatively be correlated to heterochronic processes (e.g., peramorphosis in the lineage towards *S. vandokkumi*; see Tsai & Fordyce, 2014), data on different ontogenetic stages would be needed to test any such hypothesis.

**Figure 8:**
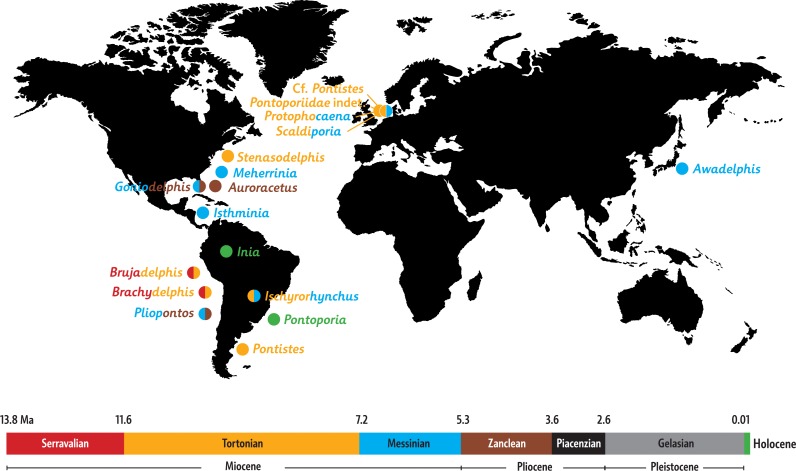
Paleobiogeography of extant and extinct inioids. World map with the Neogene and Holocene inioid records in the North and the South Atlantic Ocean, North and South Pacific Ocean, North Sea, and South American freshwater riverine systems. Colors indicate geochronological age for each individual taxon, with two colors used for taxa with uncertain ages. Sources for ages are provided in the main text, as well as taken from Morgan (1994), Cozzuol (2010), Geisler, Godfrey & Lambert (2012), and Murakami (2016).

**Paleobiogeography**: When mapping the known extinct and extant inioids it becomes clear that radiation and migration patterns are not yet resolved (Fig. 8). A single marine pontoporiid species, the coastal *Pontoporia blainvillei*, survives in the South Atlantic, whereas extant iniids (of the only surviving genus *Inia*) are presently restricted to South American freshwater ecosystems. Both are the remnants of a taxon-rich past, of which the oldest members were thought to originate from late Middle Miocene (Serravalian) deposits of the western coast of South America (Lambert et al., 2017). However, geological ages for the lower levels of the Pisco Formation are still debated and the inioid-bearing strata most likely date from the early Late Miocene (Tortonian) (Bianucci et al., 2016b; Di Celma et al., 2017). The South Pacific inioids *Brachydelphis* and *Brujadelphis* may thus not be significantly older than several inioids from the North Atlantic realm. Either way, the Central American Seaway appears to have been an important path for migrations of inioids between the Pacific and the North Atlantic. Considering the high number of Late Miocene taxa in the North Atlantic and adjoining seas, this region must have been an important center of diversification for inioids during the Late Miocene. The overall lack of fossils from Piacenzian and Gelasian strata and their low number in Zanclean strata is striking (bearing in mind the surviving extant *Pontoporia* and *Inia* in the South Atlantic and in the Amazon river systems) and might be partly due to fossilization bias. However a Pliocene extinction of inioids in the North Atlantic may coincide with the arrival and early radiation of delphinids (true dolphins) in this region (Whitmore Jr, 1994; Whitmore Jr & Kaltenbach, 2008; Bianucci, 2013).

More precise ages for different taxa from the North Atlantic realm and better resolved phylogenetic relationships between fossil inioids (including family attributions) are necessary to provide final clues about the region of origin and the possible dispersion routes of the superfamily and its two families Iniidae and Pontoporiidae.

**Figure 9:**
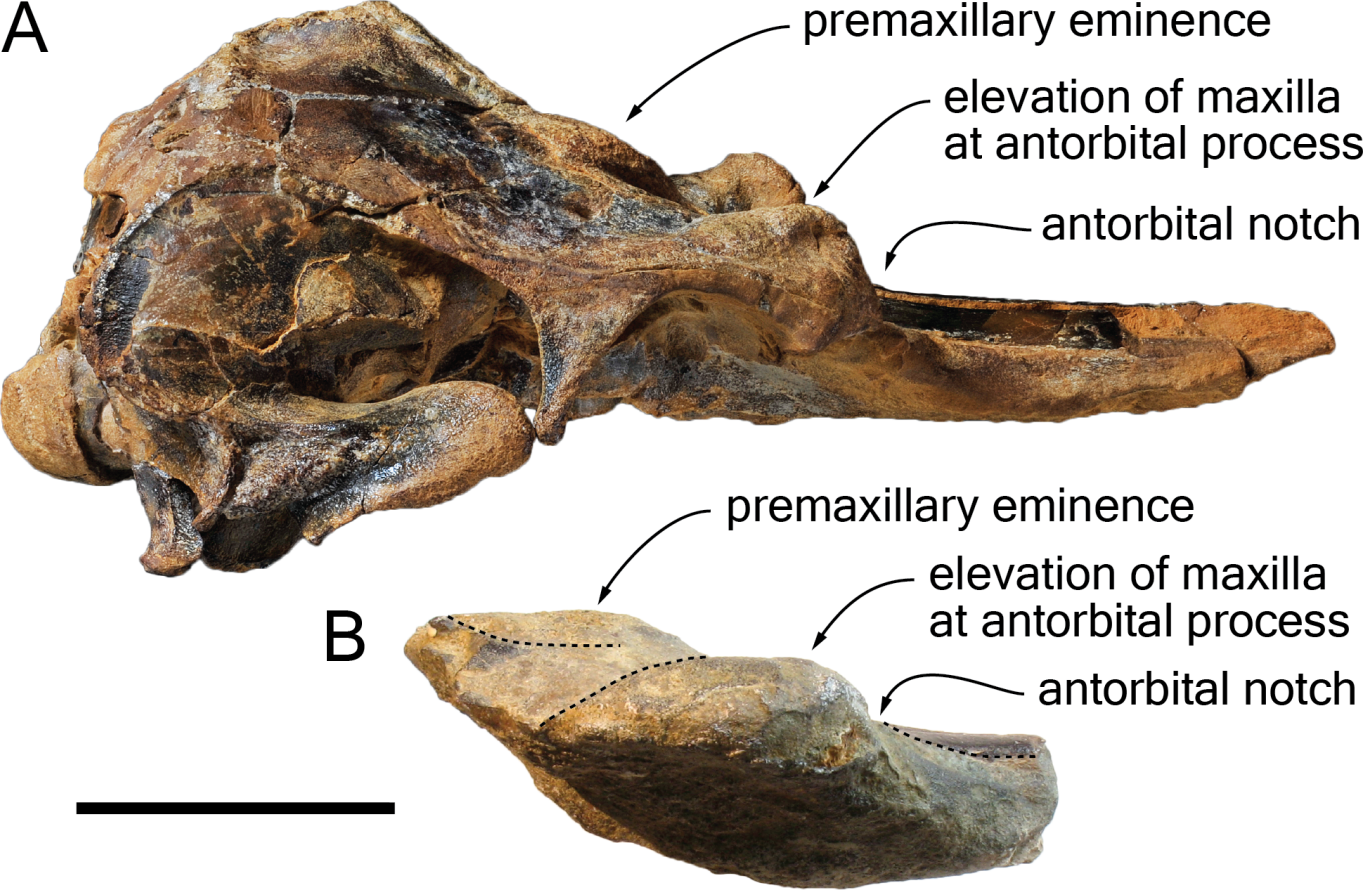
Comparison of the cranium for the pontoporiids *Brachydelphis mazeasi* and *Protophocaena minima*. (A) cranium of *B. mazeasi* MNHN PPI 266, Late Miocene of Cerro la Bruja, Pisco Basin (Peru), in right lateral view; (B) fragment of cranium of *P. minima* IRSNB M.2303, late Neogene of Steendorp (Belgium), in right lateral view. Note the similarities at the level of the antorbital region. Scale bar equals 50 mm.

**Content of the subfamily Brachydelphininae**: The subfamily Brachydelphininae was originally erected to mostly emphasize the differences in cranial proportions between the extremely short-snouted *Brachydelphis mazeasi* and the other pontoporiids (Muizon, 1988b). Later, the other short-snouted pontoporiid *Protophocaena minima* was tentatively proposed as a second member of that subfamily (Lambert & Post, 2005). Although a broad morphological phylogenetic analysis of odontocetes revealed these two genera as closely related, no sister-group relationship was found (Geisler, Godfrey & Lambert, 2012). The subsequent description of a new species of *Brachydelphis* with a significantly longer rostrum weakened somehow the diagnosis of the subfamily (Gutstein et al., 2009; Lambert & Muizon, 2013). Later cladistic analyses revealed unstable relationships between these taxa (Lambert et al., 2017), a problem most likely due to the fragmentary state of the *P. minima* specimens (lacking among others the highly phylogenetically informative ear bones). Other apomorphic characters for the subfamily Brachydelphininae are: a swollen, dorsally and laterally exposed lacrimal, and a deep and narrow antorbital notch (Lambert & Muizon, 2013). The swollen lacrimal causes an abrupt dorsal elevation of the bordering maxilla from rostrum base to the antorbital region. Although the lacrimal itself is not preserved in the holotype of *S. vandokkumi* and in known specimens of *P. minima*, the maxilla can be observed medial to the antorbital notch on the said specimens, and no marked elevation is present. This might indicate the absence of a large and swollen lacrimal*.* However, a new specimen of *P. minima* from the late Neogene of Belgium (IRSNB M.2303) displays a markedly thickened antorbital region reminiscent of the condition in *Brachydelphis* spp. (Fig. 9). Furthermore, the lacrimal and antorbital notch of the Danish pontoporiid specimens MGUH 1910-274 and 1922-168 are preserved and show exactly the condition present in *Brachydelphis* spp. Refraining from naming these fragmentarily preserved crania, Pyenson & Hoch (2007) did neither assign them to *Brachydelphis* or *Protophocaena*, based on their alleged symmetry (cranial symmetry being considered diagnostic for Pontoporiinae relative to Brachydelphininae by Muizon, 1988b). However, estimating the degree of asymmetry proves to be tricky in fragmentary crania, especially if the supraoccipital shield and basicranium are not preserved, and the validity of (more or less) facial symmetry as a diagnostic character is still debated (Gutstein et al., 2009).

A tentative inclusion of the Danish specimens and *P. minima* in the Brachydelphininae should not be ruled out. As a consequence, some members of this subfamily may lack a premaxilla-nasal contact (as observed in MGUH 1910-274; Pyenson & Hoch, 2007).

## Conclusions

1. The description of the new species *S. vandokkumi*, from Late Miocene to earliest Pliocene marine deposits of The Netherlands further increases the past morphological disparity of the family Pontoporiidae, especially for the facial region of the cranium.
2. The new taxon further demonstrates the relatively high diversity of inioids in the North Atlantic realm during the Late Miocene—earliest Pliocene. The origin of this radiation (either North Atlantic or southeast Pacific) is still to be determined.
3. The pontoporiid genera *Brachydelphis*, *Protophocaena*, and *Scaldiporia* are morphologically distinct. More complete specimens of the North Sea pontoporiid taxa are needed to test this hypothesis within a phylogenetic analysis.

##  Supplemental Information

10.7717/peerj.3991/supp-1 Supplemental Information 1 Dinoflaggelate cysts Palynological content of the sediment sample taken from the cerebral cavity of *Scaldiporia vandokkumi* NMR 9991-12018.

10.7717/peerj.3991/supp-2 Supplemental Information 2 Consensus tree

10.7717/peerj.3991/supp-3 Supplemental Information 3 Matrix

10.7717/peerj.3991/supp-4 Supplemental Information 4 Molecular constraint

## Institutional abbreviations

CMM: Calvert Marine Museum, Solomons, Maryland, USA
IRSNB: Institut Royal des Sciences Naturelles de Belgique, Brussels, Belgium
MACN: Museo Argentino de Ciencias Naturales, Buenos Aires, Argentina
MGUH: Geological Museum, University of Copenhagen, Copenhagen, Denmark
MNHN: Muséum National d’Histoire Naturelle, Paris, France
MUSM: Museo de Historia Natural, Universidad Nacional Mayor de San Marco, Lima, Peru
MSM: Museo di Storia Naturale dell’Università di Pisa, Calci, Italy
NBC: Naturalis Biodiversity Center, Leiden, The Netherlands
NMB: Natuurhistorisch Museum Boekenberg, Antwerp, Belgium
NMR: Natuurhistorisch Museum Rotterdam, Rotterdam, The Netherlands
OMB: Oertijd Museum, Boxtel, The Netherlands
TM: Teylers Museum, Haarlem, The Netherlands
USNM: National Museum of Natural History, Smithsonian Institution, Washington, D.C., USA
ZMA: Zoological Museum Amsterdam, Amsterdam, The Netherlands

## Additional Information and Declarations

### Data Availability

The following information was supplied regarding data availability: The holotype of *Scaldiporia vandokkumi* is stored in the Natural History Museum of Rotterdam, the Netherlands, with collection number 9991-12018.

### New Species Registration

The following information was supplied regarding the registration of a newly described species: Publication: urn:lsid:zoobank.org:pub:B5D0467C-E361-4278-9A8C-937B0DD11C39 *Scaldiporia*: urn:lsid:zoobank.org:act:B287E0AB-C75C-48C5-94C9-E364F2850FC1 *Scaldiporia vandokkumi*: urn:lsid:zoobank.org:act:5430C68D-303F-4CBB-9082-51FA4B43E872.
