# Supplementary material for: Scaldiporia vandokkumi, a new pontoporiid (Mammalia, Cetacea, Odontoceti) from the Late Miocene to earliest Pliocene of the Westerschelde estuary (The Netherlands)
Source: PeerJ. 2017 Nov 1;5:e3991. doi: 10.7717/peerj.3991 (PMC5671118; doi:10.7717/peerj.3991)
Supplement: Supplemental Information 1 — Palynological content of the sediment sample taken from the cerebral cavity of Scaldiporia vandokkumi NMR 9991-12018. [file peerj-05-3991-s001.docx]

| **Dinoflagellate cysts** | **n** |
| --- | --- |
| *Achomosphaera andalousiensis* | 1 |
| *Batiacasphaera hirsuta* | 1 |
| *Batiacasphaera serratum* | 1 |
| *Batiacasphaera* sp. ind. | 1 |
| *Habibacysta tectata* | 1 |
| *Invertocysta lacrymosa* | 4 |
| *Leunecysta* sp. ind. | 1 |
| *Lingulodinium machaerophorum* | 5 |
| *Melitasphaeridium choanophorum* | 4 |
| *Operculodinium centrocarpum* | 7 |
| *Operculodinium*? *eirikianum* | 1 |
| *Operculodinium israelianum* | 5 |
| *Operculodinium piaseckii* | 1 |
| *Quinquecuspis concreta* | 1 |
| *Reticulatosphaera actinocoronata* | 1 |
| *Selenopemphix armageddonensis* | 1 |
| *Spiniferites* spp. ind. | 49 |
| *Tectatodinium pellitum* | 1 |
| *Trinovantedinium*? *xylochophorum* | 1 |
| *Trinovantedinium* sp. ind. | 1 |
|  |  |
| **Acritarchs** |  |
| *Cyclopsiella elliptica*/*granosa* cpl. | 1 |
|  |  |
| Reworked dinoflagellate cysts | 6 |
