# Supplementary figures and images for: Scaldiporia vandokkumi, a new pontoporiid (Mammalia, Cetacea, Odontoceti) from the Late Miocene to earliest Pliocene of the Westerschelde estuary (The Netherlands)
Source: PeerJ. 2017 Nov 1;5:e3991. doi: 10.7717/peerj.3991 (PMC5671118; doi:10.7717/peerj.3991)

# Strict consensus tree

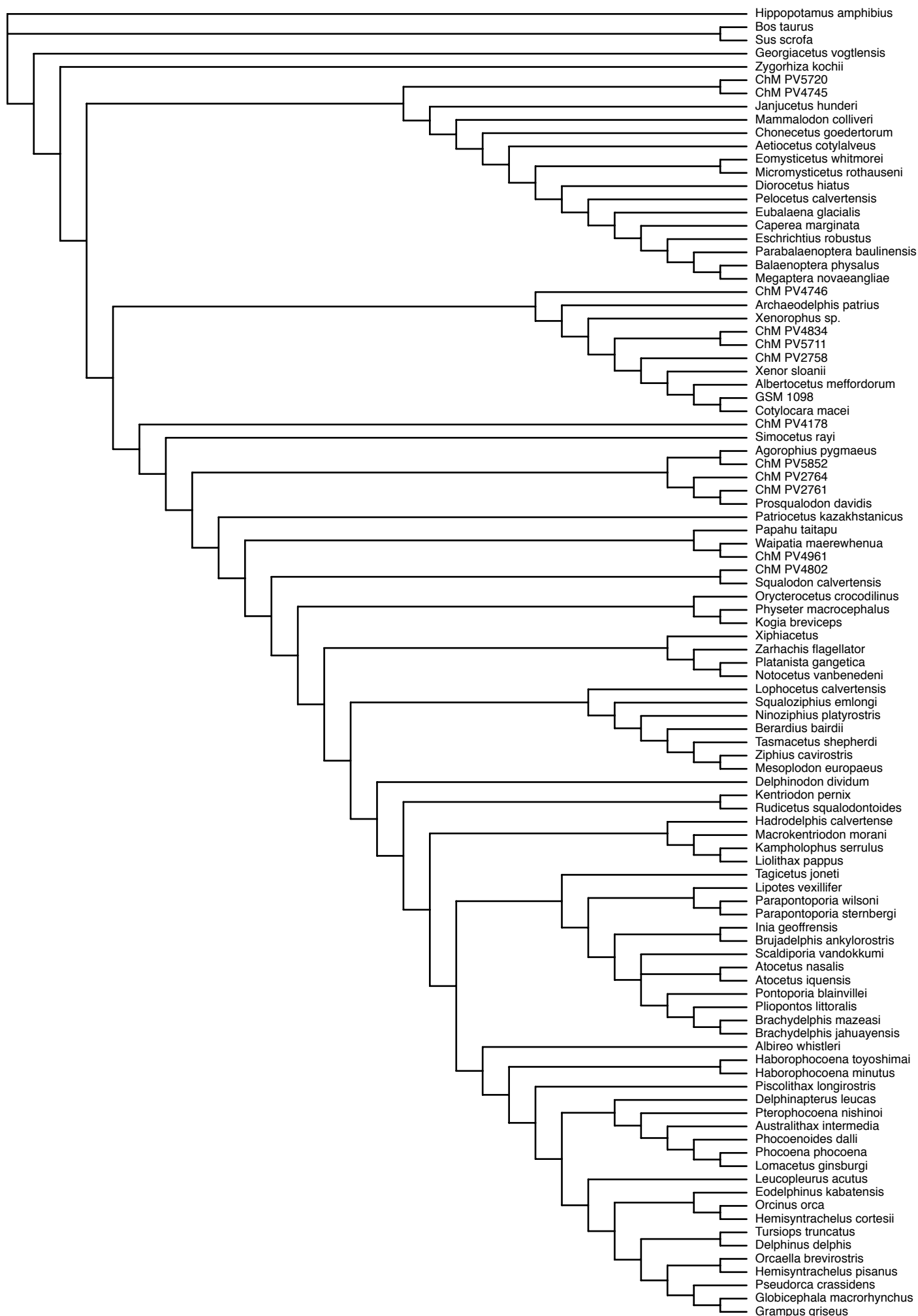

Supplement: Supplemental Information 2 [file peerj-05-3991-s002.pdf]
